# Supplementary figures and images for: Comprehensive Comparison of Effects of Antioxidant (Astaxanthin) Supplementation from Different Sources in Haliotis discus hannai Diet
Source: Antioxidants (Basel). 2023 Aug 19;12(8):1641. doi: 10.3390/antiox12081641 (PMC10451870; doi:10.3390/antiox12081641)

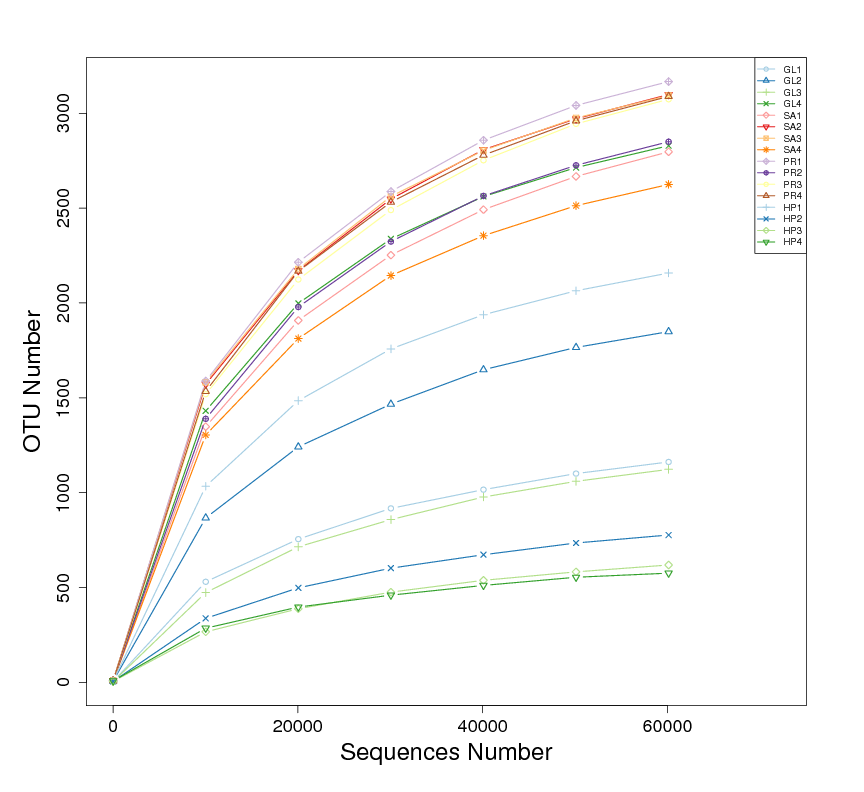

Supplement: Supplementary file 1 [file antioxidants-12-01641-s001.zip › observed_otu.png]
